# A Comparison of Hemodynamic Changes Between the Use of Etomidate and Propofol as Induction Agents for Anesthesia in Daycare Surgeries

**DOI:** 10.7759/cureus.32421

**Published:** 2022-12-12

**Authors:** Sanjeeb K Giri, Partha S Mohapatra, Laxman K Senapati, Krishna Mishra

**Affiliations:** 1 Anaesthesiology, Kalinga Institute of Medical Sciences, Bhubaneswar, IND; 2 Community Medicine, Kalinga Institute of Medical Sciences, Bhubaneswar, IND

**Keywords:** induction, heart rate, mean arterial blood pressure, diastolic blood pressure, systolic blood pressure, general anesthesia, etomidate, propofol

## Abstract

Background and objective

The development of modern anesthetic agents has made it possible to conduct pain-free surgical procedures. The role of the anesthetist in choosing a suitable anesthetic agent to provide a good anesthetic and sedative effect is very important in any surgical procedure. There is always a degree of risk involved as the hemodynamic parameters may be altered. This study aimed to compare the hemodynamic changes and respiratory effects between the use of etomidate and propofol for the induction of general anesthesia (GA) as well as to compare the side effects of both drugs in daycare surgeries.

Methods

The study was a parallel-design, randomized, double-blinded control trial conducted over a period of three years among patients undergoing elective daycare surgeries under GA. The patients were classified into two groups depending on the type of drug received: Group A or the propofol group and Group B or the etomidate group. Randomization was done by computer-generated random number generator software. A total of 174 patients were selected (87 in each group) at a ratio of 1:1. A baseline evaluation of the hemodynamic parameters was done followed by continuous monitoring.

Results

The age, weight, and gender distribution of the patients in both groups were comparable. Significant hemodynamic changes were observed following induction in Group A. The fall in systolic blood pressure (SBP), diastolic blood pressure (DBP), and mean arterial blood pressure (MABP) in Group A following induction was found to be statistically significant (p<0.00). The rise in heart rate was almost similar in both groups, with Group A demonstrating a slightly higher rate. There were fewer signs of respiratory depression in Group B. The major side effects observed after induction were myoclonus, which was more prevalent in Group B patients (21.84%), and pain at the injection site, which was observed more frequently in Group A (17.1%).

Conclusion

Based on our findings, etomidate is a drug with better hemodynamic stability and less pain at the site of injection compared to propofol. Hence, it may be a better induction agent in daycare surgeries.

## Introduction

Any surgical procedure requires the patient to be under good anesthetic effect for the surgeon to operate properly, and hence the role of an anesthetist is extremely crucial in surgery. Choosing an anesthetic agent responsibly to provide a good analgesic and sedation coverage for any surgical procedure should be the priority of any anesthetist, keeping the safety of the patient in mind. Patients who undergo certain painful procedures such as orthopedic maneuvers or drainage of an abscess often require moderate or deep sedation/analgesia for the surgeon to perform the procedures smoothly and successfully. This is performed by using sedative agents administered at a specified dose, thereby allowing the patients to maintain the airway reflexes and respond to verbal or painful stimuli [1]. In general anesthesia (GA), anesthetic agents are usually given via the intravenous (IV) route. During induction, sudden and unexpected hemodynamic changes may occur due to the physiological effects of the agents used to induce sedation and analgesia. Hence, anesthetists must use extreme caution to choose an agent with the least adverse effects and good efficacy to ensure the safety of the patients and to prevent complications [2].

Clinicians and researchers are always in search of a safer and better anesthetic agent with a quick and smooth recovery profile for the induction of anesthesia. Currently, several agents are available for the induction of anesthesia, such as propofol, thiopental, etomidate, or a combination of propofol and etomidate to name a few. Propofol is commonly used for induction in short surgical procedures whereas the action of etomidate is characterized by its hemodynamic stability and fewer side effects on the respiratory profile of the patient. In this study, we aim to compare the hemodynamic, respiratory, and other effects of the two such anesthetic agents, i.e. etomidate and propofol, during daycare surgeries with a view to determining which is the more efficacious and safer option between the two. To summarize, the study objectives were as follows: to compare the hemodynamic changes and respiratory effects between etomidate and propofol as induction agents for GA as well as to compare the side effects of both drugs in daycare surgeries.

## Materials and methods

This was a parallel-design, randomized, double-blinded control trial with patients classified into two groups at a ratio of 1:1. This study was conducted at a tertiary care center in Hyderabad over a period of three years (2016-2018) after obtaining ethical clearance from the institutional ethics committee via letter no. DNBT-60/2015. The study included patients scheduled to undergo elective daycare procedures (aged 18-65 years), with an American Society of Anesthesiologists (ASA) classification of I and II [3], and those who provided written informed consent for participation. Patients previously diagnosed with hypertension, known cases of coronary artery disease (CAD) or cerebrovascular (CVD) disease, obese patients with BMI >35, and those with known cases of preoperative hypotension were excluded from the study. The sample size was calculated to be 87 for each group using the following formula [4]:

n_1 _= (Z_α_ +Z_β_)^2^[rθ_1_ (1 - θ_1_) + θ_2_ (1 - θ_2_)]/(θ_1 _- θ_2_ - δ)^2^

Here, α is the significance level for the one-sided test (one-sided superiority hypothesis), β is the power of the test, θ1 is the expected success proportions of sample one (propofol), θ2 is the expected success proportions of sample two (etomidate), δ is the superiority margin set (0.1), r is the ratio of the sample size of group two to group one, and n is the sample size of each sample. Randomization was done by computer-generated online random number generator software. A total of 174 patients were selected and classified into two groups at a ratio of 1:1. Separate syringes were used for the preparation of the drugs to be used in the study for each participant. The procedure was conducted by a designated anesthesia resident to ensure that blinding was thoroughly maintained throughout the study. Both the investigator as well as the patient were unaware of the type of drug administered. The coded syringes were prefilled with either of the drugs in particular dosages (etomidate: 2 mg/ml; propofol: 1% 10 mg/ml). The syringes were prefilled to contain 20 ml for blinding purposes so that there was no visual difference between the prepared syringes. The first group (Group A: propofol group) included patients who received propofol 1 mg/kg bolus followed by 0.5 mg/kg every three minutes as needed for sedation. The other group (Group B: etomidate group) included patients who received etomidate 0.1 mg/kg, followed by 0.05 mg/kg every three to five minutes as needed.

A detailed pre-anesthetic evaluation comprising factors such as the history of previous medical or surgical illnesses if any, previous anesthetic exposures, allergy to any known drug in the past; and other baseline investigations including blood, X-ray of the chest, and airway examination for patency were done after obtaining written informed consent from the study participants. The patients were kept nil per oral (NPO) for almost six hours before the surgery. A record of the preoperative vital parameters in the form of baseline recordings of the pulse, blood pressure (BP), and oxygen saturation were taken for each patient. Patients underwent cardiac, BP, and pulse oximeter tests according to standard guidelines in the operation theatre. Baseline values were recorded and an IV line with an 18-gauge (G) cannula was given. All the patients were pre-medicated with fentanyl 2 µg/kg, glycopyrrolate 4 µg/kg, and ondansetron 15 µg/kg given by slow IV 20 minutes before induction. Patients were then randomized to receive either propofol 1 mg/kg bolus followed by 0.5 mg/kg every three minutes as needed for sedation or etomidate 0.1 mg/kg followed by 0.05 mg/kg every three to five minutes as needed. It was made sure that the hemodynamic parameters [heart rate (HR), systolic blood pressure (SBP), diastolic blood pressure (DBP), and mean arterial pressure (MABP)] were monitored and documented throughout the surgery. To compare the induction characteristics, the statistical analysis was done for five minutes only as the duration of action of the induction dose was five minutes, following which the adjuvant drugs exhibit their own action on the hemodynamic profile of the patient. Randomization was achieved by using a sequentially numbered sealed envelope containing the group allotment. The use of supplemental oxygen during the process was at the discretion of the treating physician.

The collected data were coded and entered into a Microsoft Excel spreadsheet and were subsequently analyzed in detail using SPSS Statistics v. 21 for Windows (IBM Corp., Armonk, NY). Descriptive statistics were used and the data were presented in the form of mean ±SD, whereas proportions were represented with a 95% confidence interval. The Chi-square test, paired t-test, and Student’s t-test were used as tests for significance wherever applicable. A p-value <0.05 was considered statistically significant.

## Results

The hemodynamic parameters in the present study were compared in terms of baseline values recorded while preparing the patient for the surgery (six hours before surgery) and values after induction with the agents (either propofol or etomidate). The Group A patients comprised those who received propofol as the induction agent for GA whereas Group B comprised those who received etomidate. Table 1 presents the basic characteristics of the study participants.

**Table 1:** Basic characteristics of the study participants SD: standard deviation

Basic characteristics			
Parameter	Propofol/Group A (n=87)	Etomidate/Group B (n=87)	P-value
Age, years, mean ±SD	35.09 ±11.27	36.2 ±11.23	0.486
Weight, kg, mean ±SD	61.38 ±11.67	64.13 ±13.64	0.171
Gender			
Male, n (%)	39 (44.8%)	36 (41.4%)	0.78
Female, n (%)	48 (55.2%)	51 (58.6%)	

**Figure 1:**
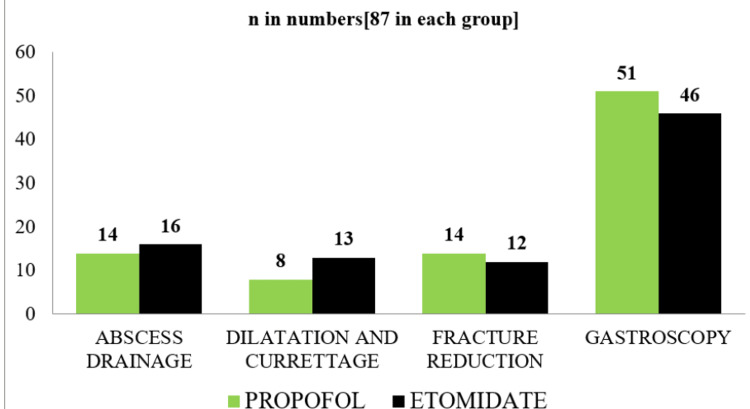
Distribution of study participants according to the type of surgical procedure

The mean age was 35.09 ±11.27 years in Group A and 36.28 ±11.23 years in Group B (p=0.486). The body weight of patients in Group A was between 41 and 100.5 kg with an average body weight of 61.38 ±11.67 kg, while in Group B, it was between 39 and 99 kg with an average body weight of 64.13 ±13.64 kg (p=0.171). Thus, the age, weight, and gender distribution of the patients in both groups were comparable, which shows that the patients of similar age, sex, and weight were enrolled in the study under the two groups. Figure 1 shows the distribution of study participants according to the type of daycare surgical procedure planned under GA.

As depicted in the figure above, the procedures conducted in both groups were almost similar, further emphasizing the fact that the two groups are comparable. Table 2 shows the hemodynamic changes observed six hours before surgery (baseline data) and after induction with the specific drugs.

**Table 2:** Hemodynamic changes observed in both groups SD: standard deviation; GA: general anesthesia

Hemodynamic parameter	Propofol/Group A (n=87)	Etomidate/Group B (n=87)	P-value
	Mean ±SD	Mean ±SD	
Systolic blood pressure, mmHg			
Baseline	131.61 ±11.084	132.43 ±11.531	0.635
After induction of GA	108.84 ±108.84	121.53 ±11.976	0.00
Diastolic blood pressure, mmHg			
Baseline	85.63 ±7.13	86.26 ±8.57	0.598
After induction of GA	70 ±6.916	79.62 ±8.21	0.00
Mean arterial blood pressure, mmHg			
Baseline	100.96 ±8.45	101.66 ±9.404	0.604
After induction of GA	82.95 ±7.878	93.59 ±8.934	0.00
Heart rate, beats/minute			
Baseline	79.2 ±10.53	78.79 ±9.91	0.79
After induction of GA	81.38 ±10.89	79.9 ±11.05	0.37

We identified that there was a fall in SBP, DBP, and MABP following induction with propofol in Group A patients when compared to patients in Group B who were administered etomidate. These differences were found to be statistically significant (p=0.00 for all). Our findings indicated that hemodynamic changes were more significantly associated with the use of propofol as the inducing agent. The rise in heart rate in Group A patients was slightly higher than that of Group B patients, but this difference was not found to be statistically significant. The factors related to respiratory changes/depression after induction are presented in Table 3.

**Table 3:** Indices depicting respiratory changes/depression in both the groups

Clinical signs of respiratory depression	Propofol/Group A (n=87), n (%)	Etomidate/Group B (n=87), n (%)	P-value
Increase in supplemental oxygen requirement	17 (19.54%)	14 (16.09%)	0.552
Use of bag and mask	6 (6.9%)	4 (4.6%)	0.515
Airway repositioning	10 (11.5%)	10 (11.49%)	1
Stimulation to induce breathing	12 (13.79%)	9 (10.34%)	0.648
Fall in saturation to <90%	10 (11.5%)	8 (9.2%)	0.619

**Figure 2:**
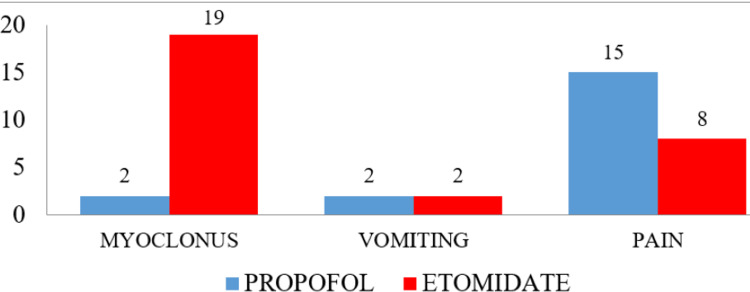
Side effects observed after induction in both groups (n=87 in each group)

It was observed that Group A patients showed slightly more clinical signs of respiratory depression compared to patients in Group B, but this difference was not found to be statistically significant. Hence, both drugs have similar effects on respiration after induction. Figure 2 depicts the side effects observed in both groups after the use of the induction agents.

Nineteen (21.84%) patients in Group A reported side effects of the drug, whereas, in Group B, 29 (33.33%) patients reported side effects. The number of patients developing myoclonus was two (2.3%) in Group A and 19 (21.84%) in Group B (p=0.000). This difference was highly statistically significant, indicating a much higher incidence of myoclonus in the etomidate group. The number of patients having pain at the injection site was 15 (17.1%) in Group A and eight (9.1%) in Group B (p=0.117); this difference was not found to be statistically significant.

## Discussion

The present study involved subjects with a mean age of 35.09 ±11.27 years in Group A and 36.28 ±11.23 years in Group B. All the patients belonged to either ASA I or ASA II classes. Similar participant details were reported in a study conducted by Karki and Singh [2]. The present study compared the effects of propofol and etomidate in daycare surgeries. Other studies have reported similar effects of the drugs in laparoscopic cholecystectomy, endoscopic retrograde cholangiopancreatography (ERCP) in obese patients, and gastrointestinal endoscopy [5-7]. The fall in SBP, DBP, and MABP in Group A was found to be statistically significant in the present study, which aligns with the findings of studies by Karki and Singh, Jain and Tichkule, and Sodhi [2,8,9]. We observed a slight increase in heart rate following induction in patients of Group A compared to Group B; however, this was not found to be statistically significant. A study by Kumar reported a significant increase in heart rate following induction with propofol, which was also found to be statistically significant [10]. However, a study by Alipour et al. [11] reported that intraocular pressure and heart rate significantly decreased following induction with propofol than etomidate, which contrasts with our findings.

The indices depicting respiratory depression were comparable in both groups and were not found to be statistically significant. A study by Eames et al. reported a greater respiratory depression with etomidate compared to propofol, which was also found to be statistically significant [12]. Similar results with respect to SBP and DBP have been reported by Patel et al. and Meena et al. [13,14]. However, a study by Miner et al. reported a different finding: they found that etomidate and propofol resulted in similar rates of sedation, subclinical respiratory depression, hypoxia, apnea, and clinical events related to respiratory depression; propofol had a higher rate of procedural success than etomidate and none of these differences resulted in clinically significant adverse events. It was reported that both of these medications were similarly safe for use in procedural sedation [1]. Similar results have been reported by Rocchio et al. [15].

The side effects of both drugs were mostly similar, but a greater incidence of myoclonus was reported in Group B. The major complaint with the use of propofol was pain at the injection site. Similar results have been reported in the study by Kumar [10], but the rate of incidents reported was higher compared to our findings. The results reported by Tudu et al. contrast with the present study as none of their study participants reported any side effects following the induction of GA with either etomidate or propofol [5]. The findings of Govardhane et al. are in line with our study with respect to hemodynamic changes and side effects of both drugs [16].

We did not investigate the association between etomidate and adrenal suppression and this parameter was not included in the study, since it was not feasible to determine the effect of etomidate on adrenal behavior with the administration of a single bolus dose. Further studies may be designed to evaluate this effect of etomidate. Also, we believe that there is scope to use BIS monitoring and the Ramsay sedation scale in further research on the current topic.

## Conclusions

Based on our findings, etomidate is a better anesthetic induction agent compared to propofol. It demonstrated greater hemodynamic stability and less pain on being injected. The drawback observed with etomidate was the incidence of myoclonus. Etomidate has shown better acceptability among patients compared to propofol as pain during the injection is less than that with propofol. Hence, etomidate can be preferred over propofol as the induction agent in patients with existing cardiac illnesses in whom maintaining stable hemodynamic parameters is very important during induction for a favorable outcome. It can be used as the drug of choice for daycare surgeries.
